# Sensor Networks in the Low Lands

**DOI:** 10.3390/s100908504

**Published:** 2010-09-10

**Authors:** Nirvana Meratnia, Berend Jan van der Zwaag, Hylke W. van Dijk, Dennis J. A. Bijwaard, Paul J. M. Havinga

**Affiliations:** 1 Pervasive Systems, University of Twente, P.O. Box 217, 7500 AE Enschede, The Netherlands; E-Mails: b.j.vanderzwaag@utwente.nl (B.J.Z.); h.w.vandijk@utwente.nl (H.W.D.); P.J.M.Havinga@utwente.nl (P.J.M.H.); 2 Inertia Technology, P.O. Box 217, 7500 AE Enschede, The Netherlands; E-Mail: dennis@inertia-technology.com (D.B.)

**Keywords:** wireless sensor networks, environmental sensor networks, body sensor networks, structure sensor networks, transport sensor networks, participatory sensor networks

## Abstract

This paper provides an overview of scientific and industrial developments of the last decade in the area of sensor networks in The Netherlands (Low Lands). The goal is to highlight areas in which the Netherlands has made most contributions and is currently a dominant player in the field of sensor networks. On the one hand, motivations, addressed topics, and initiatives taken in this period are presented, while on the other hand, special emphasis is given to identifying current and future trends and formulating a vision for the coming five to ten years. The presented overview and trend analysis clearly show that Dutch research and industrial efforts, in line with recent worldwide developments in the field of sensor technology, present a clear shift from sensor node platforms, operating systems, communication, networking, and data management aspects of the sensor networks to reasoning/cognition, control, and actuation.

## 1. Introduction

Over the past decade, research and industrial communities have witnessed a major paradigm shift in the area of communication and computation. The widespread use and availability of various wired and wireless communication mediums, miniaturisation and decreasing size and cost of computing and sensing devices, as well as increasing power and accuracy of them have enabled design and development of spatially extended networks of multi-variable intelligent sensor arrays to monitor complex phenomena. A new generation of these networks is the wireless sensor network (WSN), which typically consists of a large number of low-power sensor nodes usually equipped with a wireless transceiver, a small microcontroller, an energy power source and multi-type sensors such as temperature, humidity, light, heat, pressure, sound, motion, *etc*.

The multidisciplinary nature of wireless sensor networks brings research, industry, and end-users in a close collaboration. As a new and emerging field, the very first years of developing WSNs have been dedicated to making the hardware platform mature enough to be used for real experimentation and implementation. What started as a prototyping platform, is now commercially available in various forms, shapes, sizes, and functionality running various operating systems (e.g., TinyOS [1] or AmbientRT [2]).

In this paper, we present the evolution of sensor networks in the Netherlands from 2002 till 2010 and highlight maturity stages and topics addressed over the past decade. By providing an overview of related topics, projects, and initiatives, we aim to show the past trends and identify the new directions and challenges ahead. To do so, we look at the evolution of sensor networks in the Netherlands from various angles including research topics and challenges, different types and topologies of sensor networks, and sensor network applications.

## 2. Sensor Networks in the Netherlands

Looking at the evolution of sensor networks in the Netherlands, we can distinguish three main directions: (i) sensing and hardware platforms, (ii) communication and networking, and (iii) middleware. Additionally, testbeds and experimental platforms as well as social aspects of ambient intelligence and sensor networks have always been discussion points. Various projects such as Smart Surroundings [3], Embedded WiSeNts [4], e-SENSE [5], and SENSEI [6] have paid special attention to user acceptability and effects of technology on society at large.

### 2.1. Sensing and hardware platform

**Figure 1.**
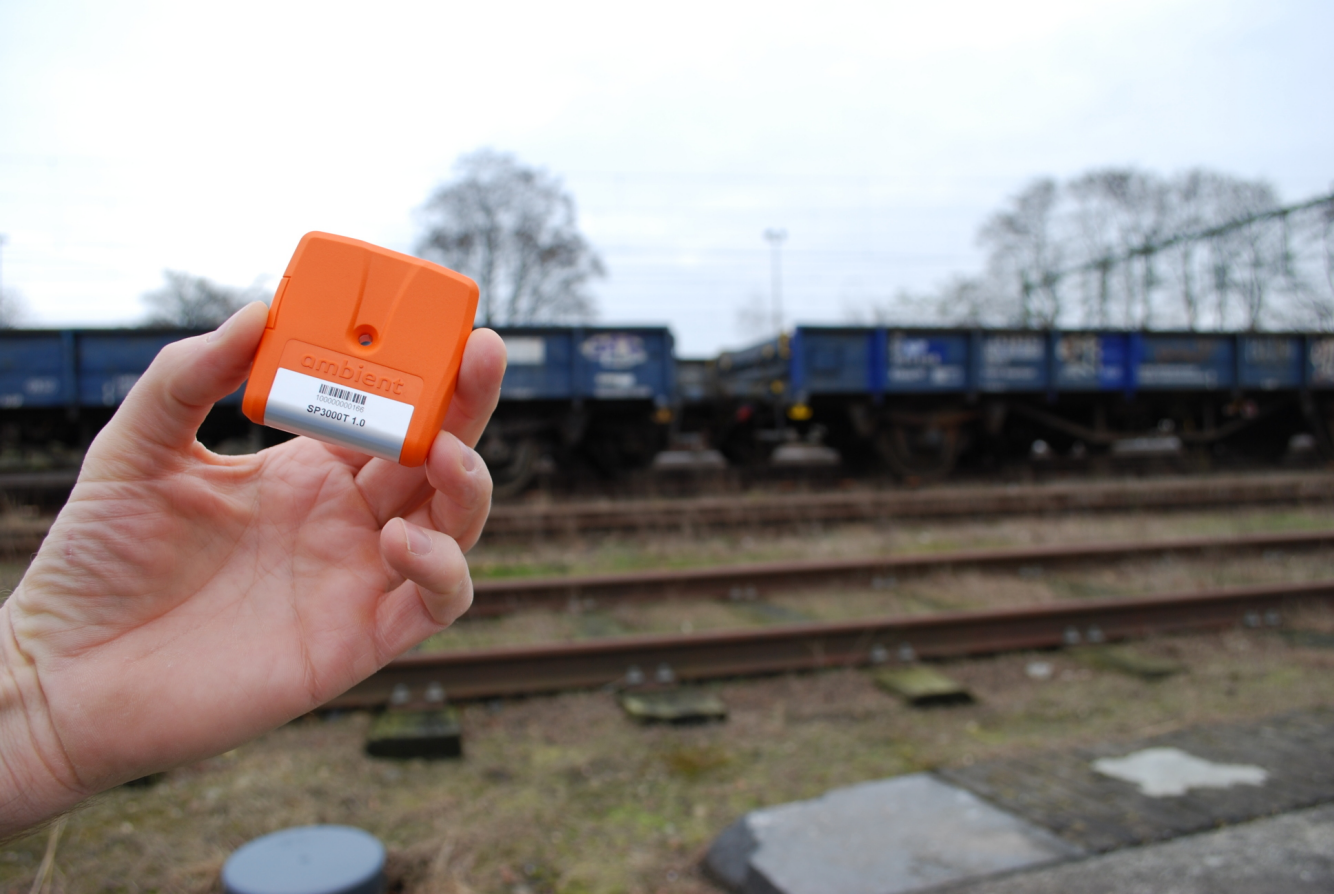
Smart Point: sensor node of Ambient Systems [8].

Research and industrial development of wireless sensor networks started with design and development of hardware platforms. In 2002, the Netherlands initiated and led the first European project on wireless sensor networks, namely EYES [7]. The EYES project aimed at developing an architecture and hardware platform required for building self-organising and collaborative sensor networks. In the context of the EYES project and in terms of sensor node hardware platform, the Netherlands has made major contributions to the field through design and development of the Ambient sensor node platform [8] (shown in Figure 1), the AmbientRT operating system [2], LMAC [9], and T-Mac [10].

### 2.2. Communication and networking

Projects such as EYES [7], MobiHealth [11], Consensus [12], CoBIs [13], Featherlight [14], Awareness [15], and WASP [16] have developed networking and communication solutions for wireless sensor networks. The idea of the MobiHealth project was to fully support mobility of patients while their vital signs were measured and were made available to medical teams for monitoring and feedback. Through a programmable mobile phone or any other personal device, collected health information could be sent to a central place, where a medical team would observe the health conditions of the patient and send their feedback via the same device. The Consensus project aimed at enabling collaboration between wireless sensor nodes through design of cross-layer optimised communication and networking protocols. The focus was on investigating effects of networking and communication layers on the data layer and vice versa. The cross-layer optimisation approaches of the project have enabled design and development of, among others, energy efficient data aggregation and querying mechanisms. The CoBIs project proposed a new approach for business processes by close integration of monitoring physical entities into enterprise applications. This project not only addressed integration of heterogeneous hardware platforms but also proposed new reliable routing and dissemination protocols as well as simple rule-based engines for wireless sensor networks. The focus of Featherlight was on the development of energy-efficient, distributed, light-weight, and computationally cheap networking and distributed data processing solutions for wireless sensor networks. The Awareness project made connection via WiFi/GPRS/UMTS to a back-end server, through which health professionals could remotely monitor health status of their patients. The WASP project focused mainly on self-organisation aspects of the wireless sensor networks. The project established a strong link with the hardware platform as self-organisation and reconfigurability aspects of the network require a thorough understanding of hardware behaviour.

### 2.3. Middleware

**Figure 2.**
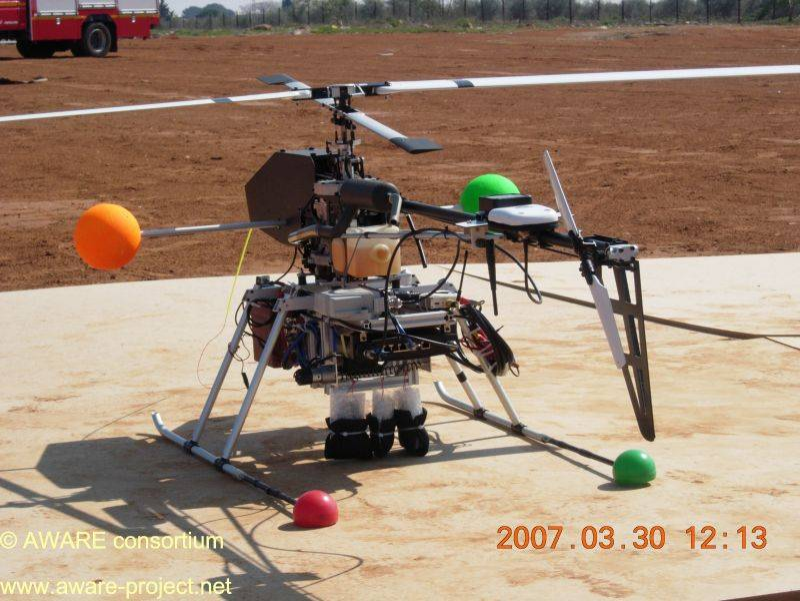
Example of aerial flying object used in the AWARE project [17].

Middleware has also received much attention through projects such as e-SENSE [5], AWARE [17], SENSEI [6], and iLAND [18]. The main contribution of the e-SENSE project (Capturing Ambient Intelligence for Mobile Communications through Wireless Sensor Networks) was the design of key enabling technologies for ambient intelligent systems through very low power, highly efficient and distributed middleware solutions for wireless sensor networks. The AWARE project aimed to design and develop a middleware platform to enable collaboration between ground-based wireless sensor and actuator networks including mobile vehicles and people with aerial flying objects. An example of such aerial flying objects can be seen in Figure 2. The project focused on harsh and difficult-to-access environments without communication infrastructure. The SENSEI project creates an open, business driven architecture that fundamentally addresses the scalability problems for a large number of globally distributed Wireless Sensor and Actuator Network (WS&AN) devices. It provides necessary network and information management services as middleware functionality to enable reliable and accurate context information retrieval and interaction with the physical environment. By adding mechanisms for accounting, security, privacy and trust, it enables an open and secure market space for context-awareness and real world interaction. The iLAND project [18] develops middleware and associated technologies for frequent and deterministic reconfigurations in service oriented architectures. The Dutch consortium implements an early warning system based on opportunistic networking.

#### Localisation

In the area of localisation, the Netherlands has major contributions (and patents) through projects such as EYES [7] (WSN-based localisation), Smart Surroundings [3] (WiFi-based localisation), and Relate [19] (ultrasound-based localisation). Localisation in both static and mobile sensor networks have been addressed in various research papers such as [20–22]. One of the results of the Smart Surroundings project has been design of algorithms for motion detection and WiFi-based localisation based on fusing sensor readings across different sensing technologies [23]. The Relate project (Relative Positioning of Mobile Objects in Ad hoc Networks) investigated relative positioning of mobile objects to determine their spatial arrangement free of surrounding infrastructure, as opposed to a more commonly used external infrastructure installed in the system’s environment to acquire and manage position and location information. The project addressed some fundamental research challenges that such an approach implies. These include how to obtain relative positions in ad hoc systems, how to reason about dynamic spatial arrangements, and how to provide application support across different domains. COOL [24] is a development platform enabling users to combine data from various sensors into new services. Example use cases include an office scenario, to track people and to manage flexible work stations, and a training scenario to monitor location and body characteristics like heart beat and oxygen saturation. COOL processes data centrally.

#### Context-awareness

The Smart Surroundings project [3] is one of the first Dutch projects to lay down a flexible architecture and enabling mechanisms for ambient intelligence and context awareness. The all-inclusive approach of the project spanned the entire spectrum ranging from scenarios and use cases, requirements and architecture down to networking and communication. Various settings including office, personal health care, and home context awareness were addressed. The Amigo project [25] used open middleware to provide intelligent user services for home care and safety, home information and entertainment applications. Amigo allows easy plug-in of sensors for capturing data, aggregation of raw sensor data to meaningful attributes (e.g., location) and context inferencing to determine higher-level context like activity and relative position of objects.

#### Resource management

Projects such as Featherlight [14], Smart Surroundings [3], and e-SENSE [5] have proposed mathematical analyses [26] as well as algorithmic approaches [27] for distributed resource management integrated with publish/subscribe middleware to cope with the resource-constrained nature of wireless sensor networks.

### 2.4. Testbeds

The Netherlands has also a strong presence in the area of large-scale testbeds for wireless sensor networks. KonTest [28], a wireless sensor network testbed at Vrije Universiteit Amsterdam, consists of a 60-node indoor wireless sensor network testbed, distributed among six office rooms. The testbed has been in use since 2008 as the main evaluation platform for novel wireless sensor network protocols and systems. WISEBED [29] aims to provide a multi-level infrastructure of interconnected testbeds of large-scale wireless sensor networks for research purposes, pursuing an interdisciplinary approach that integrates the aspects of hardware, software, algorithms, and data. This will demonstrate how heterogeneous small-scale devices and testbeds can be brought together to form well-organised, large-scale structures, rather than just some large network. It will allow research not only at a much larger scale, but also in different quality, due to heterogeneous structure and the ability to deal with dynamic scenarios, both in membership and location. The wireless sensor network testbed of the University of Twente, spanning through offices, the Smart eXperience lab (SmartXp) [30], and the entire campus is at this moment under development. One of the results of SENSEI [6], being realised at this moment, will be a Pan-European test platform, enabling large-scale experimental evaluation of project results and execution of field trials—providing a tool for long-term evaluation of WS&AN integration into the Future Internet.

## 3. Sensor Network Topologies

Applications involving WSNs are very diverse and involve one or a combination of various types of sensor networks. By looking at the evolution of WSN related projects in the Netherlands and around the world, we can identify five types of wireless sensor networks:

- **Environmental sensor network (ESN):** Environmental sensor networks are the very first type of wireless sensor networks. Traditionally, environmental sensor networks were solely deployed for monitoring and data collection purposes. These networks have been deployed in a large variety of sites spanning through sea, air, and land.
  Environmental sensor networks are often large scale, static, non-dense, and are deployed in harsh and unattended environments. Heterogeneity of ESNs has been more in terms of different types of sensor nodes (e.g., resource-limited nodes, routers, gateways) rather than types of sensors used in deployment. The more recent networks of this type extend their single-hop communication to also support multi-hop communications and tend to have more than one sink node. Due to their large scale and harsh unattended deployment area, energy efficiency, long network life-time, and security have always been the major concerns of these types of sensor networks.
- **Body sensor network (BSN):** Body sensor networks (BSNs) are types of sensor networks consisting of very few wireless sensor nodes on or around a living being’s body integrated with one more powerful device such as a personal device (e.g., smart phone). Until very recently, monitoring vital signs, tracking, and data collection have been the main objectives of these sensor networks. Normal practise has been centred around off-line analysis of collected data from body sensor networks by experts and providing feedback (mainly in the field of health and well-being).
  Compared with ESNs, BSNs are small scale, heterogeneous (in terms of different types of sensors) and require single-hop communication. Due to the fact that various personal information can be collected by these networks, both security and privacy are major concerns. Unlike ESNs, in BSNs energy consumption is a secondary concern while reliable data processing and timely feedback are of higher importance.
- **Structure sensor network (SSN):** Structure sensor networks of medium to large numbers of wireless nodes usually attached to buildings (e.g., office), structures (e.g., bridges), infrastructure (e.g., rails) or deployed in specific venues (industrial sites). Compared with ESNs that are almost always outdoors, SSNs may be deployed both indoors and outdoors and combine several environments simultaneously. Examples of the latter include restricted access and public spaces of a single building or aboveground and underground sections of a single railway. In terms of security, SSNs are often more security sensitive than ESNs and require protection mechanisms against both physical and electronic attacks. Due to their difficult deployment, energy efficiency and long network life-time are of high importance for SSNs. Similar to most traditional environmental sensor networks, SSNs are often static. Structure sensor networks may be both single and multi hop (depending on their scale) and are often heterogeneous (in terms of both sensor nodes functionality and type of sensors).
- **Transport and logistics sensor network (TSN)** Transportation means such as cars, trucks, and trains, all have built-in wired sensor networks. Over the past few years, many efforts have been directed towards wireless communication and networking between these transportation vehicles. To this end, various communication standards such as IEEE 802.11p (for vehicle to vehicle communication) have been set. Each individual vehicle can be seen as a sensor node, which locally observes its own status while it also monitors its surroundings.
  Depending on their applications, TSNs may be either in the form of network of vehicles or a combination of vehicle networks and SSNs (e.g., warehouse logistics). In the former case, energy efficiency is less important since the vehicle itself can provide the required energy. Also, in the former case, the network itself is mobile and it requires multi-hop communication due to the fact that accident and emergency information should be disseminated the furthest as possible.
- **Participatory sensor network (PSN):** Recent advances in mobile technology have extended functionality of the mobile phones to the degree that making and receiving phone calls are considered to be their very basic tasks. Mobile phones are becoming more and more equipped with sensors (e.g., GPS, accelerometer, gyroscope, camera) and different types of connectivity mediums (bluetooth, wifi, GSM, *etc*.). This combination makes the mobile phone and in fact people carrying them a valuable source of collecting and transmitting information. Information collected by people through their mobile phones can range from personal health conditions and their trajectory to environmental conditions and pictures of the area in which they move around.

Behind each of the above network types, there is a large hidden set of requirements and offered functionalities. Therefore, it is worth highlighting distinguishing factors between these network types and identifying a set of common high-level features. Although it seems difficult to clearly distinguish between the above-mentioned sensor network types, some factors such as scale, network life time, support for mobility, density, and diversity enable distinguishing them. Table 1 represents such distinction.

## 4. Sensor Network Application Areas

Sensor network research and development is often targeting specific application areas. In the following, we give a brief overview of such application areas and we indicate per area which aspects have been or are expected to become focus points in sensor network research and development in the Netherlands.

### 4.1. Transport and logistics

**Figure 3.**
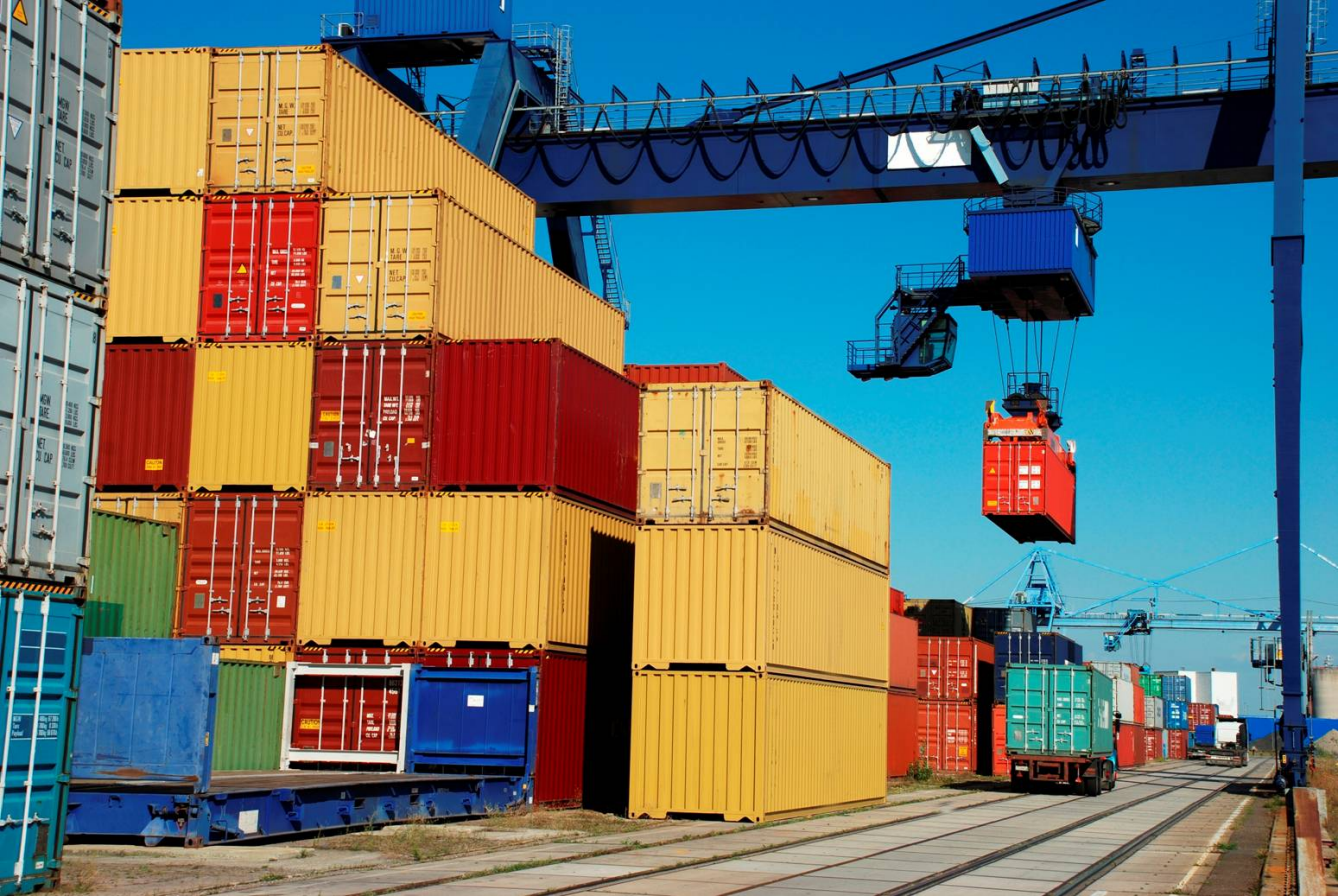
Container terminal in transport and logistics.

For transport and logistics, goods can be monitored while they are in warehouses and when they are in transit. Figure 3 shows an example of such application. Especially in the cool chain market, it is important to optimise the quality of perishable products (like food and pharmaceutical items) by ensuring optimal storage and transport conditions. These products often also need to comply with regulations, including showing what the conditions were during storage and transport. In addition, assets can be tracked when they enter or leave certain areas. This can be used to track returnable transport items like roll containers and also for finished product items that should not leave the yard or warehouse undetected. A relevant project in this area using wireless sensor networks is the Free project [31], which proposes an all-inclusive way of energy management for wireless sensor networks for transport and logistics applications. On the one hand, it proposes energy-efficient communication and data-processing mechanisms to reduce energy consumption of the network operations, while on the other hand, it investigates feasibility of energy harvesting technology to produce and replace the spent energy. The ATMO project [32] is another related project, which aims to increase reliability and availability of traffic sensor data. The project includes every thinkable source of data ranging from road cameras and induction loops to in-car navigation systems. An obstacle for this centralised data fusion is the fact that technology must most prominently have a legal and privacy origin. A related challenge is to formulate the required reliability for specific applications like traffic routing for environmental and economical reasons.

Ambient Systems [8], Almende [33] and Nedap Specials [34] are active with wireless sensor solutions in this market. The WSN topology used in this field are TSN in trucks, cars and ships, and SSNs in warehouses and storage yards. Competing products are data loggers that have to be individually read out after transport or storage.

### 4.2. Health care and wellbeing

In hospitals and revalidation centres, nowadays solutions become available that patients can use in their home environment for tele-monitoring. The WSN topology used in this field is mostly the BSN, sometimes augmented with sensor readings from training objects or environmental sensors in the building (SSNs). In the MobiHealth project [11], a mobile device with attached body sensors was used to monitor the patient condition remotely. Successful results of this project have enabled establishing a spin-off company called MobiHealth [35]. The MobiHealth system was also used in the Freeband Awareness project [15]. The IS-Active project [36] focuses on devise of a real-time and person-centric health-care solution for patients with chronic conditions. The project goes beyond centralised and offline systems by developing online and distributed data processing, activity recognition, and reasoning mechanisms to run on wireless sensor nodes attached to patient bodies and reason about collected data locally instead of sending raw data to a medical team. The Ambient Living with Embedded Networks (ALwEN) project [37] deals with the combination of BSNs, ESNs, SSNs, and Telemedicine to implement a novel approach to zeroth, first and second line care and to address the widely recognised fact that care must be organised in a patient-centric manner. The project has set as its noble course: technology to assist people in their day to day chores. Its target is to contribute to a more independent life of the elderly and people suffering from a physical impairment. Some companies active in this field are Xsens [38], Nedap Specials [34], Almende [33] and Roessingh [39].

### 4.3. Fitness and sports

**Figure 4.**
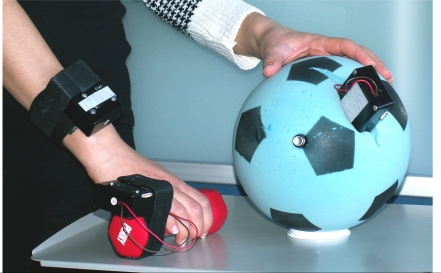
Interaction of body sensor network with objects.

Motion sensors such as accelerometer, gyroscope, and compass provide means to quantify the level of training as well as performance in sports activities. Real-time feedback is essential for quickly assessing the training efficiency, as well as preventing overuse injuries. Xsens [38] is active in this field with sensors wired to a mobile device (wired BSN), while Inertia Technology [40] is active in this field with a wireless BSN as well as offering real-time feedback on interaction with training objects. An example of such interaction is illustrated in Figure 4.

### 4.4. Environmental monitoring

Environmental monitoring applications of WSNs in the Netherlands include soil, space, and underwater ecosystems monitoring.

**Figure 5.**
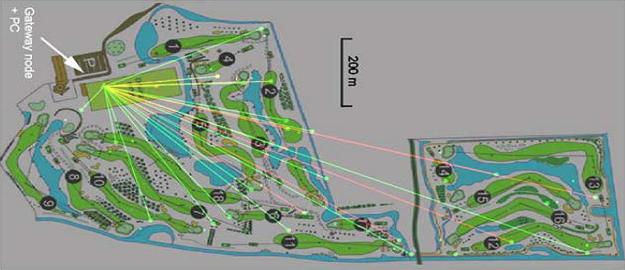
Wireless sensor network used for monitoring soil moisture of a golf course [41].

To continuously measure soil moisture at different locations and depths, wireless sensor nodes have been deployed in a golf course [41]. The new underground infrastructure based on wireless sensor network technology has shown potential to be used for various applications ranging from golf field monitoring to precision agriculture. Fast and easy installation of the wireless sensor network, (near) real-time monitoring capability of a large space, and availability of data through an easy web interface are attractive features offered by this project. Figure 5 shows the layout of the Almkreek golf course in the Netherlands, where a wireless sensor network has been used for monitoring soil moisture.

The RIVM [42] is a governmental organisation for public health and environment, which owns several measurement networks. The national sensor network air quality (LKM) [43] monitors various aspects of air quality. This network has 22 sensor points in urban areas and about 30 more in rural areas, covering the entire country. Typically measured values include small particles, ozone (O_3_), carbon oxides (CO*_x_*), nitrogen oxides (NO*_x_*), acids (SO_2_, NH_3_), metals, *etc.* Other sensor networks of the RIVM include soil quality measurement network (LMB), radioactivity measurement network (NMR), soil water measurement network (LMG), and sound. Sound measurements are limited to a few (10) selected points like railway crossings and highways.

Other examples of air quality measuring network and sound measurement network are the networks of DCMR [44] in the Rotterdam area.

**Figure 6.**
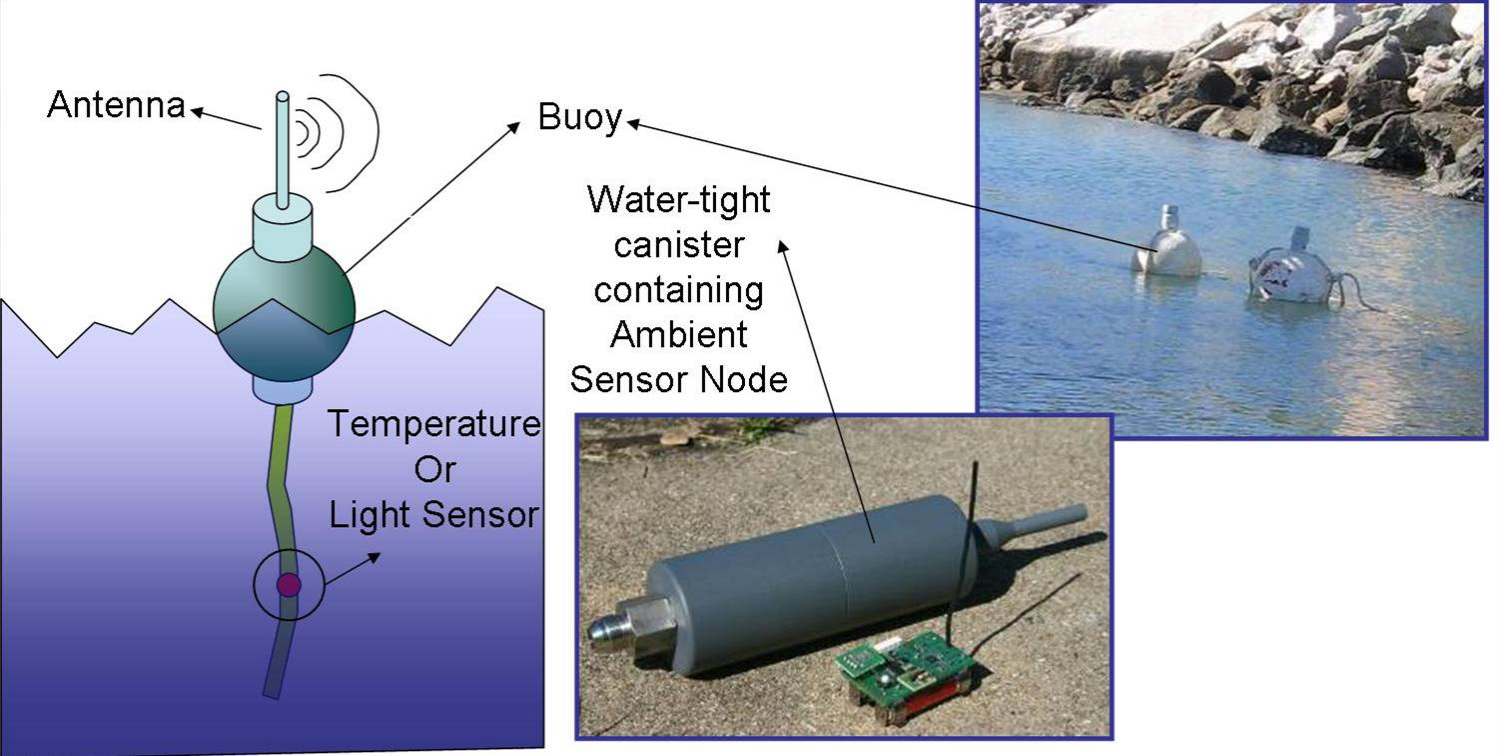
Wireless sensor network deployed in the Great Barrier Reef [46].

A joint effort between the Netherlands and the Australian Institute of Marine Science (AIMS) [45] has lead to monitoring the Great Barrier Reef in Australia. By setting up a large-scale wireless sensor network in Davies Reef, which is approximately 80km northeast of the city of Townsville in North Queensland, to monitor various environmental parameters on the reef, scientists at AIMS intend to use the collected data to study coral bleaching, reef-wide temperature fluctuations, and the impact of temperature on aquatic life and pollution. Figure 6 shows the wireless sensor node used in monitoring the Great Barrier Reef [46].

Various efforts have also been directed toward making sensor data available for environmental monitoring applications. For instance, the RGI-189 project [47] involves the development of a sensor web-based approach combining earth observation and in situ sensor data to derive typical information offered by a dynamic web mapping service (WMS). A prototype was developed which provided daily maps of vegetation productivity for the Netherlands with a spatial resolution of 250 m. Daily available MODIS surface reflectance products and meteorological parameters obtained through a Sensor Observation Service (SOS) were used as input for a vegetation productivity model.

**Figure 7.**
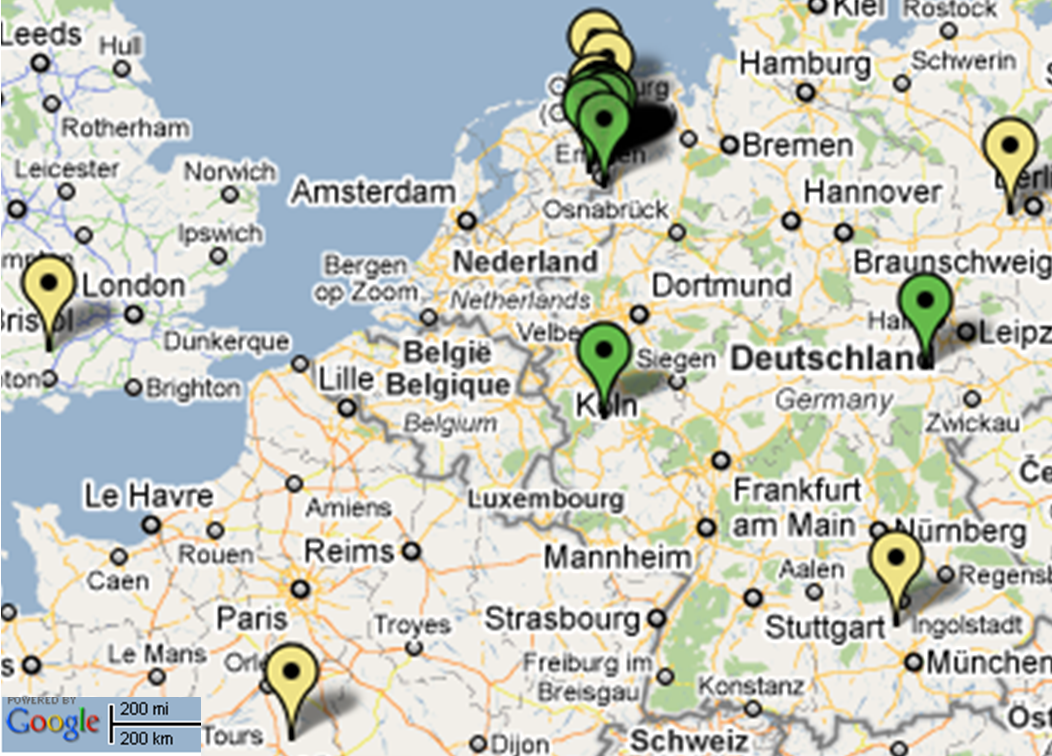
Geographical spread of LOFAR stations as of 22 July 2010 (green: completed; yellow: under construction) [49].

Lofar [48], the LOw Frequency ARray, is a multi-purpose sensor array currently being assembled in the Northeast of the Netherlands and spreading over the whole country and over all of Europe. The geographical spread of the currently existing Lofar stations is depicted in Figure 7. Lofar consists of a hierarchically structured sensor system, which is a aperture synthesis array composed of phased array stations. Sensor fields form a station that select one of multiple beams (phased array); these beams are transferred over a high speed (glass fibre) wide area network to a central processing unit, which convolves data from various stations to create the aperture synthesis array. In this configuration, the Lofar system is used for deep space observation.

The Lofar infrastructure comprises 18 Core station fields, 18 Remote station fields, 10 Geo-Remote station fields with Geophones and Microbarometers, and 8 International station fields. Its base line length ranges from 100 m to 1,500 km. The Geophones and Microbarometers, measuring infrasound, enable seismic monitoring of the earth crust up to 5–10 km of depth [50]. The recorded measurements are fused off-line with meteorological measurements from surrounding weather stations. This is done in close cooperation with the national meteorological institute KNMI [51], which controls a large network of weather stations each comprised of a variety of sensors.

### 4.5. Agriculture

Using the Lofar data transmission infrastructure, LoFAR-Agro project [52] has made measurements of the micro-climate in potato crops. This information was used to improve the advice on how to combat phytophtora (a fungal disease in potatoes) within a crop, based on the circumstances within each individual field. The phytophtora project made use of Motes, which consist of a radio transmitter and a sensor board. The radio works at a frequency of 433 MHz and can cover distances of up to 15–30 metres. The sensor part of the Mote measures air pressure, temperature, relative humidity and illumination. Because the humidity of the soil is a major factor in the development of the micro climate, a number of sensors that measure soil humidity was also included in each field.

**Figure 8.**
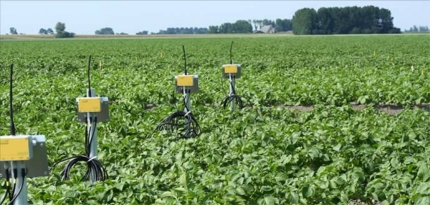
Example use of the wireless sensor network in agriculture by WaterSense [53].

Figure 8 illustrates an example use of the wireless sensor networks in agriculture done in the WaterSense project [53]. WaterSense is a test bed for a decision support system (DSS) on water management. The project installs a wireless sensor network with about 100 stationary sensing points that measure water levels in various drains and the deeper soil as well as salinity of water. The DSS fuses additional data from weather reports and water levels in canals and lakes. Furthermore a large amount of models is available to the DSS. The future aim is to create precision control for water and fertilisation. This will increase a crop’s health and yield.

### 4.6. Structure monitoring

This application area focuses on sensing the condition within or outside structures like buildings and bridges. This field qualifies for a WSN topology of its own, namely SSN. Because of the fixed installation of infrastructure nodes, the SSN can also be used for real-time location monitoring, e.g. by Ambient Systems [8]. SOWnet [54] and Automatic Signal [55] created GuArtNet to protect items of art in museums or in possession of art collectors.

The IJkdijk [56] is an artificial sample dike (100 metres long and 6 metres high) to understand the physical process of dike bursts due to abnormal situations. Sensor technology is being developed to act as an early warning system, which can autonomously, possibly supported with visual inspections, predict dike bursts accurately. The Livedijk [57] is a realistic experiment in which the results of IJkdijk have been applied. The sensor network comprises a wide range of glass fibre connected sensors: 32 water tension meters, 53 inclination meters, 34 water and soil temperature sensors, 1 water level sensor, 1 air pressure sensor, 1 ambient temperature sensor, 1 relative ambient humidity sensor, 4 inertial (acceleration) sensors, approx. 50 wave height measurement points on the dike core and 1328 in-fibre temperature sensors. Initial measurement frequency is set to 1 every 5 minutes, which can be increased in case of suspected anomalies. The actual system combines weather and sea reports with the measurements from the dike sensor network.

### 4.7. Entertainment

The entertainment field includes film, TV, games, and advertising. Current sensor usage is for motion capturing, for which the BSN is the most suitable topology. For location tracking also the SSN topology in combination with a carried wireless node could be used. A strong player in this field is XSens [38], that for instance uses motion capturing to support the creation of animated video. The Amigo project [25] had also entertainment as one of its target areas and envisioned sensors at home for this.

### 4.8. Domotics

Home automation or domotics comprises applications such as climate control, lighting and ambience adaptation, energy saving systems, security systems, and so on, in a domestic setting. Nedap Specials [34] uses Zigbee nodes for indoor localisation, which can be used as input for other domotics applications. PTC rm&s company [58] specialises itself in data transfer and communications by means of a self-forming and self-healing wireless mesh network, in which all connections are automatically made and maintained in a mesh structure. This network enables sensing at each wireless node, which is used for home and building automation as well as metering and energy management. Eaton/Holec [59] develops its xComfort product line with wireless switches and dimmers for home appliances like light, window shutters and door. It uses hand-held remote controllers (868 MHz band). In addition it provides a home controller which can act as a gateway to the home system. Philips Lighting [60] is yet another example of a company offering smart solutions for domestic lighting control.

### 4.9. Industrial safety

**Figure 9.**
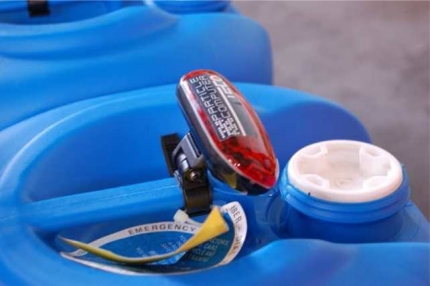
Wireless sensor node used for an industrial safety application [61].

Wireless sensor networks can offer a valuable solution to industrial safety through continuous monitoring, timely detection of hazardous situations. By generating alarms or taking smart actions whenever and wherever dangerous situations occur, catastrophical situations can be prevented. The CoBIs project [13] investigates this matter through design and implementation of a distributed, service-oriented enterprise system, which incorporates the latest advances in WSN technology. The field tests of solutions designed in the project were carried out at a chemical plant of BP in Hull, UK for identification of storage of hazardous substances and continuous monitoring of the chemical plant conditions [61]. Figure 9 shows the sensor node used for the field tests. The SENSEI project [6] also addresses industrial safety applications by proposing distributed online activity recognition solutions to run in wireless sensor networks to ensure that workers in a plant follow safety regulation procedures and to inform them when hazardous situations are about to happen.

## 5. Current and Future Trends

**Figure 10.**
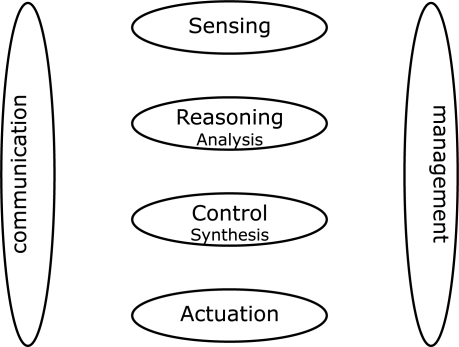
Components of sensor networks.

In the above sections we demonstrated the evolution of Sensor Networks in the Netherlands. Current sensor networks go beyond traditional environmental monitoring systems which were focussing on data collection only. The trend in sensor networks involves a paradigm shift towards reasoning, control, and actuation, and therefore more *interaction* with the environment. Figure 10 illustrates the different components, which together make the vision of sensor networks come true.

Traditional sensor networks concentrated on monitoring activities, sensing physically observable parameters from the field and bringing the data ashore. The actual data analysis, handling, and control were taking place in the backbone using powerful machines. Many modern sensor networks incorporate simple actuators such as LEDs or alarm generators. For these networks, the actuation is very close to the sensing point and inclusion of more complex actuation and control mechanisms in wireless sensor networks is by no means trivial or mature.

The sensor network field has investigated versatile deployment by introducing wireless, in particular RF, communication. Many projects have taken into account various aspects of communication, routing, and networking technology. At the same time, the field realised that radio transceivers are sensors and actuators themselves, and can be used as such. A notable example is the use of radio signal strength for autonomous localisation algorithms. Here the same device is used for both communication and sensing.

New projects such as CLAM (CoLlAborative eMbedded networks for submarine surveillance, starting in 2010) and SeaSTAR (Underwater Monitoring Platform, also starting in 2010) are exploring new communication media and have sensor networks monitor and communicate underwater.

As more and more sensor networks will surround people, human–sensor interfaces will become more and more important for future systems. This calls for in-depth research of human–sensor interfaces.

Sensor networks operate in the immediate environment of the system under observation (in-situ). Increasing the density of these networks, induces research in the direction of instrumenting the network with reasoning and control capabilities, simply because the network will become congested if no actions are taken. Increasing the scale calls for more attention to management aspects such as scheduling and parallel executions. Structuring observations hierarchically is yet another point of attention.

Future sensor networks will be diverse in their hardware, software, and mobility. Integrating different components and their protocols in a generic way is an open research problem. Interesting emerging application domains become available by combining different types of sensor networks, e.g., the effective integration of in-car, car-to-car, and car-to-curb networks. The necessary integration of reasoning and control is further complicated by different mobility patterns of the nodes.

Programming models, ontologies, and analysis techniques are required for expanding sensor networks. The organic behaviour of sensor networks makes it a real challenge to predict the behaviour of the system as a whole, while diversity in sensor networks requires semantic alignment; for one to maintain effective data mining.

Sensor networks are maturing. Their pervasive deployment calls for a whole line of research. In case of use for monitoring critical infrastructure, issues such as dependability, scalability, availability, safety and security are significant bottlenecks if not addressed adequately. In addition, maintenance and life time of large-scale sensor networks becomes an issue, which requires ease of deployment through flexible middleware. Sustainable technology and long term operation are fundamental requirements; these can for instance be achieved through energy harvesting, intelligent technology, and energy-aware applications.

In summary the field of sensor networks is changing rapidly. Although we can borrow concepts from existing distributed systems there are many challenges remaining. We expect to find novel applications in underwater monitoring, traffic control, multimedia distribution, critical infrastructure monitoring, social networking, green and sustainable (infra)structures, and public safety and security.

## 6. Enablers

Looking at the history of wireless sensor networks in the Netherlands, one can see enormous contributions of the EU and governmental funding agencies to make the Netherlands one of the leading countries in the field of sensor networks. With proven success of wireless sensor network applications and coming about of more commercial products, new enablers and initiatives are expected to appear. In what follows, the major past and current enablers will be mentioned.

### 6.1. Governmental initiatives

- **National funding programs** are one of the important enablers of evolution of sensor network technology in the Netherlands. Many of the above mentioned projects have been financed by programs such as the NWO Programme for Research on Embedded Systems & Software (PROGRESS) [62], STW [63], IOP Gencom [64], BSiK [65], and PointOne [66]. However, many more projects have been realised through EU funds.
- **IIP sensor networks** [67] is one of the fourteen ICT innovation platforms in the Netherlands, that brings together all stake holders in the field of sensor networks. Its mission includes the application of intelligent sensor networks in selected socially relevant themes, for which it will offer solutions and increase economical prospect. Further, the platform will represent the collaborative vision of stake holders for the further development of knowledge, infrastructure, and technology of intelligent sensor networks.
- **Sensor city** [68] is an initiative of the province of Drenthe and the city of Assen to create a tangible city-wide platform for sensor system applications. The first applications comprise a measurement network for evaluating the sound landscape of a city and an intelligent mobility system to guide traffic dynamically. Processing and analysis are mainly centralised.

### 6.2. Industrial initiatives

- **Sensor universe** [69] is a platform for sensor technology that brings together industry, education, research and government. Sensor universe supports initiatives in the Northern part of the Netherlands to develop sensor technology, that is new developments and extension into international projects.
- **Target** [70] is an expertise centre that is building a sustainable economic cluster of intelligent sensor network information systems in the Northern part of the Netherlands, aimed at data management for very large amounts of data. Prominent scientific research groups and innovative businesses jointly develop and improve complex and scalable data systems. The starting point here is the Target paradigm: full integration of large-scale data processing, archiving and analysis. In these experimental surroundings, the Target model is developed into actual market applications, and participants in follow-up projects will develop further products and services.

### 6.3. Research initiatives

- **CTIT** [71] is one of the multidisciplinary research institutes of the University of Twente within the area of telematics and information technology. One of the strengths of CTIT is bringing together research and industry on the one hand, and technology and social aspects on the other hand. Wireless and sensor networks is one of the strategic research orientations of CTIT.
- **SmartXp** [30] is a user experience lab at the University of Twente that offers a flexible environment for full-scale experiments in a realistic setting. This laboratory closes the gap in technology development between prototyping and in-context deployment. For sensor networks, realistic settings include interference from radio transmitters, the opportunity to control environmental settings like light, and the interaction with other technologies as well as with humans. Running experiments include meta-data management for sensor networks, bluetooth-based localisation, energy-driven environmental monitoring, and emergent event monitoring in a car park. Further, the lab houses a Motelab-based test bed for education and research on wireless sensor networks.
- **INCAS^3^** [72] is a research institute that creates high-quality knowledge in the field of sensors and sensor systems by working together with industry and the scientific community. INCAS^3^ specialises in cognitive sensor systems. A particular context is set by large-scale sensor networks, in which a huge amount of data cannot be processed centrally. In-network processing will mitigate the data streams. The actual application of cognitive sensor systems is an important objective of the institute. Currently three application areas have been identified: environmental monitoring (air quality), health and sports, and radiation detection. The latter may help to analyse soil structures based on natural radiation from radioactive elements.

## 7. Conclusions

The Netherlands’ contribution to the field of sensor networks is both enormous and diverse. Sensor network application areas in which the Netherlands is an active player comprise application areas that are important for industry (transport and logistics, agriculture, industrial safety, structure monitoring), for people (health care and wellbeing, fitness and sports, entertainment, domotics), and for the environment (environmental monitoring).

Until now many efforts have been directed towards innovative technology: sensor node platforms, operating systems, communication, networking, and data management aspects of sensor networks. Thanks to these valuable efforts now commercial products and research-oriented solutions are available for sensing, communication, and networking.

It is now time for the Dutch research and industrial communities to expand sensor networks to explicitly include sensing, reasoning/cognition, control, and actuation. It is only through realisation of these aspects that the entire loop of the sensor network components can be closed and the vision of ambient intelligence, smart spaces, healthy society, and sustainable environments can be realised. To this end, scalability, dependability, reliability, robustness, energy efficiency, use of energy harvesting technology, security, and privacy are the challenges ahead.

## Figures and Tables

**Table 1.** A comparison between different sensor network types.

	Covered area			Life-time		Mobility		Density		Diversity	
	Large	Medium	Small	Long	Short	Mobile	Static	Low	High	Homogeneous	Heterogeneous
ESN	✓			✓			✓	✓		✓	
BSN			✓		✓	✓		✓			✓
SSN	✓	✓	✓	✓			✓		✓		✓
TSN	✓	✓			✓	✓	✓		✓	✓	✓
PSN	✓				✓	✓		✓	✓		✓
